# Handbook of Helminthiasis for Public Health

**DOI:** 10.3201/eid1304.070032

**Published:** 2007-04

**Authors:** Ronald Blanton

**Affiliations:** * Case Western Reserve University, Cleveland, Ohio, USA

“I'll never forget the day I read a book.” Daniel Pinkwater begins his book commentaries for National Public Radio with this Durante-Barnett tune. This came to mind when I realized I have never before read a textbook on parasitology from start to finish. I usually rely on a reference to refresh my memory about a detail of this or that life cycle or to reinforce a grant application with the number of those afflicted across the globe. Handbook of Helminthiasis for Public Health by D.W.T. Crompton and Lorenzo Savioli, however, bears reading straight through. What makes a complete reading so well worthwhile and also sets the book apart from most on either public health or parasitology is its successful marriage of these 2 points of view. This book covers the most common helminths by focusing on the parts of their biology that are most relevant to public health. Methods for rapid inexpensive surveys, international health initiatives, the economics of boreholes, and latrine design are discussed next to metacercarial development.

The focus on helminths also sets this book apart. In the first place, helminths are naturally engaging because of their ability to integrate their own complex biology with human biology and culture. Second, a significant re-evaluation is under way regarding the influence of parasitic worm infection on health. This refutes the perception in some circles that most helminthic infections are less harmful than the common cold. The first chapter of this book presents the more complete recent analyses that give a broader view of the true consequences of health.

Handbook of Helminthiasis for Public Health is structured in 3 parts: Human Health and Helminth Infection, Helminthology, and Control Interventions. Part 1 establishes the context for understanding the effects of helminth infections, the economics of these infections, and the resources required to control these infections. These are important aspects of infection because poverty, sanitation, national politics, and economic influences all contribute to the spread of helminthiasis but are often the most neglected aspects of helminth epidemiology. The book also recognizes the importance of urban settings for these infections, which is appropriate for a year during which, many projections say, the global human population becomes predominantly urban. The first chapter returns to specifics by ending with a comprehensive list of helminths that have been found to infect humans, a list that extends for 10 pages.

Part 2, Helminthology, is organized in part by phylogeny and in part by common transmission characteristics; thus, cestodes and schistosomes are presented in separate chapters while the soil-transmitted and foodborne helminths are grouped together. This section is devoid of the usual life-cycle diagrams but does a good job of describing the biology most relevant to public health surveillance and control measures. Useful diagrams are provided of parasite morphology and a large number of tables and charts about age and geographic distribution, illness rates, response to control campaigns, and drug dosages.

In part 3, Control Interventions, problems such as drug resistance, health education, assessment of health awareness in a population, the structure of latrines, and the place of helminth control in the international political arena are addressed and made concrete by reference to many specific infections covered in the preceding section. The book also offers 4 appendices: a glossary, a list of journals about helminthology and control intervention, detection methods in helminthology for stool samples, and a model framework for control of foodborne trematodes. A list of the 63 tables is provided, and the table of contents is well organized.

No book is without its faults; there are a few frank errors. Death from *Schistosoma mansoni* and *S. japonicum* is primarily a result of portal hypertension and esophageal bleeding with preservation of hepatic function, rather than hepatic failure as indicated in the book. The immunology references are old, except where current vaccine development is discussed. The book also fails at times from overgeneralization. Cysticercosis is diagnosed in the United States and Canada; it is just rarely transmitted in these countries. More abbreviations (e.g., U5MR, FBT, MDG, FECRT, KAP) should be included in the glossary or index. An abundance of useful figures and tables are provided, but more maps would be useful in Part 2 of the book.

Handbook of Helminthiasis for Public Health is very readable. The core audience, according to the jacket cover, is readers who have a public health background and workers involved in control programs. However, the book should interest parasitologists and even basic researchers who wish to understand the full context of helminth biology.

